# ‘Fear, uncertain, tired…...’ psychological distress among pulmonary hypertension patients: a qualitative interview study

**DOI:** 10.1186/s12888-024-05539-z

**Published:** 2024-02-05

**Authors:** Juxia Zhang, Yiyin Zhang, Yuhuan Yin, Yuping Feng, Rong Zhang, Hongyan Meng, Jing Wang

**Affiliations:** 1 Clinical Educational Department, Gansu Provincial Hospital, Lanzhou, 730000 Gansu China; 2 School of Nursing, Gansu University of Chinese Medicine, Lanzhou, 730000 Gansu China

**Keywords:** Psychological distress, Pulmonary hypertension, Qualitative study

## Abstract

**Background:**

Patient-centered health care for patients with pulmonary hypertension (PH) is important and requires an understanding of patient experiences. However, there is a lack of approaches to examine what's the effects and how the disease impact patients’ psychological well-beings.

**Methods:**

We conducted qualitative interviews with PH patient representatives to understand patient psychological experiences and inform patient-centered research and care. Participants were chosen from a tertiary hospital located in northwest China. 20 patients with PH who be treated at the hospital (13 participants were women, aged 18-74 years) were strategically selected and individually interviewed. We used qualitative analysis to identify themes relating to existential psychological distress that would clarify the nature of such concerns.

**Results:**

We found that patients experience tremendous psychological distress throughout the treatment process. Four categories that describe patients' psychological experiences emerged: burden of PH treatment, fear and uncertainty about the disease, frustration in social and family role, and lack of recognition of the condition.

**Conclusions:**

Existential concerns are salient in PH and involve the experience of loss and disruptions to the sense of self and relationships. Healthcare practitioners must work more in collaboration to detect patients' need for support and to develop the patient's own skills to manage daily life. The PH teams should tailor interventions to provide emotional, informational and instrumental support and guidance to patients.

## Background

Pulmonary hypertension (PH) is a kind of progressive disease, which caused by kinds of heterologous diseases and different pathogenesis, and results in persistent increase of pulmonary vascular resistance and pulmonary arterial pressure [1]. An estimated prevalence of PH at the population level is approximately 1% to 3% [2]. The clinical classification of PH distinguishes five groups: 1) Pulmonary arterial hypertension (PAH); 2) PH associated with left heart disease (PH-LHD); 3) PH associated with lung diseases and/or hypoxia (PH-LD); 4) PH associated with pulmonary artery obstructions (mainly chronic thromboembolic PH; CTEPH); 5) PH with unclear and/or multi-factorial mechanisms [3]. Although tremendous heterogeneity exists within each group, they share common features in pulmonary arterial remodeling and functional changes, which can cause laborious dyspnea, chest tightness, chest pain, fatigue and syncope [4], further leading disability, high mortality and poor prognosis, and the median survival rates are 86 and 61% at one and five years after diagnosis, respectively [5, 6].

Currently there is no cure for the disease, however, new therapeutic approaches to PH have been developed within the last decade [7]. Furthermore, the management of PH is increasingly complex, including medications administered by different routes with unpredictable degrees of adverse effects, options for invasive or surgical interventions, and considerations of cost and follow-up [8], all which requires a multifaceted, holistic, and multidisciplinary approach, with active involvement of PH patients. Despite advances in clinical, diagnostic, and new therapies, PH remains a debilitating illness that severely impact patients’ relationships with family and friends, their ability to work and exercise, and their financial security [9] which further put tremendous pressure on patients. Previous studies reported high prevalence of symptoms of depression and anxiety among PH patients [10–12]. These adverse psychological and emotional disturbances are significantly correlated with impaired quality of life [10, 13], worse disease prognosis [14–16], and shorten survival [12]. In order to improve patient care and disease management, it is important to gain a greater understanding of how individuals are affected and cope with these symptoms from patient perspective [17]. However, while most studies capture information about medical treatment and management of PH in a diverse patient population, participants may not have been able to discuss their psychological experiences most important to them in the day-to-day life. A review identifying 19 qualitative articles from Europe, North, South America and Asia, highlighted the needs for additional researches to explore the individual's perspective of experience with PH in different care facilities, and different background [18].

Streamlining the care of PH patients in daily clinical practice is a challenging but essential requirement for effectively managing PH [3]*.* Obviously, there is gap for PH management between China and developed countries. The 2021 National Health Data in China reported that of the 11,606 hospitals included, only 2.3% had specific beds for PH [19]. Moreover, right heart catheterization (RHC) is the gold standard for diagnosing and classifying PH [2]. There only 6.9% of hospitals in China were able to perform it [19]. To our knowledge, there is only one qualitative study in Asia which explored the psychiatric intervention experiences of patients with PH in Taiwan [20], and no such data in mainland China has been published.

In order to gain information on burden of PH and how PH affects individuals’ life, our research team conducted a study on “Burden of Pulmonary Hypertension in China: A Mixed-methods Study” which use qualitative and quantitative methods to describe resource consumption, related costs, treatment patterns, and quality of life, as well as mental health in adult patients with PH. The quantitative data which reported 70.09% of depressive symptoms among participates has been published [21]. Here we report the results of a qualitative study on the psychological experience of PH patients. This report should provide a vital patient perspective for informing policy regarding the psychological management of individuals, and also the survey provides an important platform to allow patients to share their experiences that are of most importance to them.

## Methods

### Study design

This research adopts phenomenological research method in qualitative research. Pay attention to the characteristics of the situation of the research object, and emphasize that reasonable explanations should be given on the basis of full understanding of a certain phenomenon. An interpretative phenomenological approach is adopted to explore the psychological experience of patients during the course of illness, aiming to understand the impact of illness on the body and mind of patients, and explain the efforts made by patients to adapt to the disease and the corresponding results.. The study was reported following consolidated criteria for reporting qualitative research (COREQ) [22].

### Setting and participates

Participants were recruited from a tertiary hospital located in northwest of China, one

large provincial level hospital. Patients were purposely sampled to include those agreed to participate with the Hospital Anxiety and Depression Scale (HADS)$>7$. The detailed eligibility criteria were described in previous study [21]. According to their gender, age, education background, we aimed for ethnic diversity within the sample. The sample size is based on the principle of information saturation, that is, the interview data are repeated, and no new themes will appear in the data analysis.

### Data collection

Based on literature, the outline of the interview was initially made according to the research objective, and subsequently 5 patients with PH were pre-interviewed. The outline was further modified accordingly which including: 1. What symptoms do you have, what psychological feelings? 2. What is the impact of the disease on your life, study, work, social life, family and future? 3. Can you talk about your current treatment ideas? 4. What is your expectation for the future?

The final semi-structured interview was conducted by two female researchers (author 1 and author 2) who had no prior relationship with the participants. Author 1 is a registered nurse with Master’s degree in Nursing and having previous experience of research into PH. Author 2, postgraduate student, has a background in sociology. In the phase of the sample selection, participants were informed about aim of the research project, and importance of the participant’s collaboration. Author 2 will be responsible for recruiting eligible participants and collecting their written informed consent. Firstly, the purpose and significance of this study were introduced to the respondents. Once consent is obtained, participants are asked to sign an informed consent form.

For patients who do not have problems with physical activity, they are invited to a quiet, well-lit room in the cardiology department, otherwise, the interview was conducted at patient’s bedside. No one else presented during interviews except the participant and the interviewers (author 1 and author 2). Interviews lasted 40 to 60 min, were digitally audio-recorded with participants’ permission, and transcribed verbatim. To ensure data confidentiality, an ID number was assigned to each participant and the transcripts were without personal information. During the interview, the researcher did not interrupt the interviewees at random, guided them to express their feelings deeply, carefully observed their facial expressions, tone of voice and body movements, and made notes. The recordings were transcribed into text within 24 hours of the interview. The final version of the interview transcripts was be returned to participants to assess any discrepancies and provide additional elucidation that may improve data accuracy.

### Data analysis

Qualitative data were extracted and analyzed thematically by two of the authors (author 2 and author 3) using NVivo software (version 12, QSR International) [23]. Data were reviewed and coded according to emergent themes in the data, related to psychological disturbance; and perceived barriers of psychological disturbance. Major themes and exemplar quotes are presented throughout the results. Ambiguities and disagreements were discussed by the authors until a consensus was reached. Then, fundamental structure was described. Finally, the research findings were returned to the interviewees and discussed the results with them. Participants' comments on the study results were obtained.

### Ethics approval

This study involved patients with PH who were treated at a tertiary hospital located in northwest of China. The research proposal was approved by the research Ethics committee of Gansu Provincial Hospital (Number: 2022-209). All study participants were given verbal and written information relating to the study aims and their involvement. Written consent was obtained and participants were given assurances concerning confidentiality and anonymity of their responses. Participants were also reassured that their care would not be affected in any way, whether or not they decided to take part in an interview, and it was made clear that they could withdraw from the study at any time.

## Results

A total of 20 patients were interviewed, including 13 females and 7 males, which were numbered P1 ~ P20. The detailed information was showed in Table 1.

**Table 1:** Demographic data of sample

ID	Age	Gender	Education	Marriage	Career	PH Type
P1	26	Male	Associate diploma	Unmarried	Teacher	Group 4
P2	74	Female	Primary School	Widow	Retired	Group 3
P3	35	Female	Junior middle school	Married	Unemployed	Group 1
P4	22	Male	Bachelor’s degree	Unmarried	Student	Group 1
P5	58	Female	Associate diploma	Married	Retired	Group 5
P6	59	Male	Bachelor’s degree	Married	Manager	Group 5
P7	44	Female	Primary School	Married	farmer	Group 5
P8	64	Male	Associate diploma	Married	Retired	Group 3
P9	33	Female	Associate diploma	Married	Worker	Group 1
P10	32	Female	Junior middle school	Divorced	Unemployed	Group 1
P11	72	Female	Illiterate	Widow	farmer	Group 5
P12	64	Female	Junior middle school	Married	Retired	Group 5
P13	69	Female	Primary School	Married	farmer	Group 2
P14	35	Male	Bachelor’s degree	Unmarried	Worker	Group 4
P15	73	Female	Bachelor’s degree	Widow	Retired	Group 4
P16	18	Male	Senior middle school	Unmarried	Student	Group 1
P17	57	Female	Primary School	Married	Worker	Group 5
P18	72	Male	Primary School	Married	farmer	Group 3
P19	62	Female	Illiterate	Married	farmer	Group 1
P20	27	Female	Senior middle school	Unmarried	Unemployed	Group 1

### Psychological experience of living with PH

**Fig. 1:**
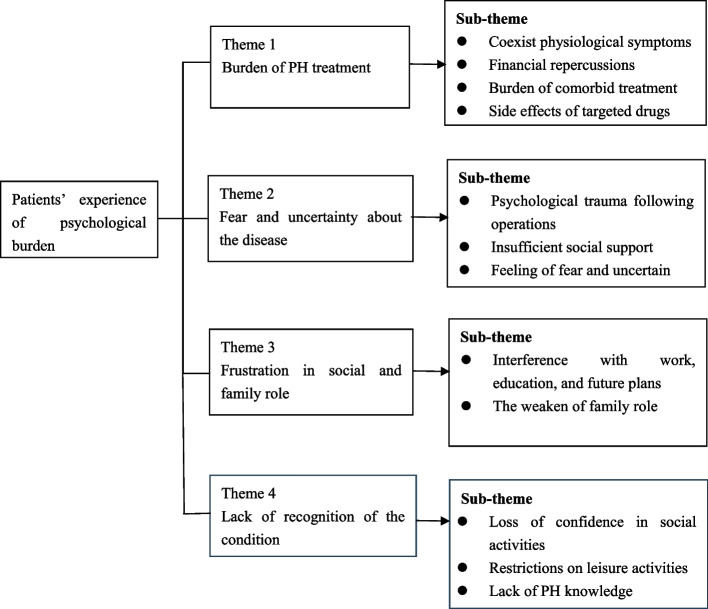
Diagrammatic of the themes and subthemes within the thematic framework

The qualitative results showed that the psychological burdens among PH patients were mainly caused by burden of disease treatment, fear and uncertainty about the disease, frustration in social and healthcare setting, and lack of recognition of the condition (Fig. 1).

### Theme 1: Burden of disease treatment

#### Physiological symptoms coexist

A range of symptoms were experienced by the interview, whereas shortness of breath and fatigue were the most commonly reported negative experiences. Respondents noted *‘I couldn't breathe with lying flat. I had to sit up and rely on oxygen all day (patient 11)’, ‘I experience breathlessness on even 300 meters walking, and I can't climb stairs (patient 17)’, ‘I feel weak and tired. I was tired and irritable. I don't want to talk to anyone (patient 1)’.* Respondents also reported other cardiac symptoms, such as dizziness, chest pain, chest congestion, and palpitation: *‘I can hear my heart beating clearly. The plop plop sound made me feel like my heart was going to jump out of my chest (patient 4)’, ‘At night, my heart was beating so fast, I was so flustered and scared (patient 20).’* Many participants were plagued with sleep disorders, with the most fundamental and immediate causes of difficulty breathing at night *‘Shortness of breath gets worse at night, which makes it impossible for me to sleep (patient 8)’.* In addition, coughing, hemoptysis, palpitations can also cause difficulty falling asleep or waking up early *‘sleeping was difficult because the hemoptysis, chest pain, chest tightness, and headache just constantly (patient 10)’, ‘At night, my heart was beating so fast, I was so flustered and scared, I couldn't sleep at all (patient 20)’.*

#### Side effects of targeted drugs

While targeted drugs relieve symptoms, they also bring side effects to patients. *‘The targeted drugs make me nausea, diarrhea, lumbago, back pain, and buttock pain (patient 3)’, ‘Riociguat can cause hypotension, and feel dizzy, the doctor told me it was normal to have tolerable side effects and I needed to keep taking the drugs (patient 15)’, ‘There was bruising and pain at the injection site with Treprostinil (patient 16)’.*

#### Burden of comorbid treatment

Those with more comorbidities tend to have worse physical conditions, more frequent hospitalizations, more difficult treatment, and take multiple medications every day. In this situation, some people may have suicidal thoughts. ‘*I suffer from COPD, hypertension, diabetes and stomach disease. I have to take a lot of medicine every day and I have to go to the hospital frequently, which is a big financial burden for my children (patient 3)’, ‘I've often thought that if I took all those pills at once, the result would be either a miraculous recovery or death, so I don't have to suffer anymore (patient 11)’.*

### Financial repercussions

Participants said they face huge direct and indirect financial burdens, including high medical and surgical costs and transportation costs Importantly, some patients suspend treatment because they cannot afford it. ‘*I spend nearly 5,000 RMB on drugs every month, which is a great financial pressure for me who is unemployed (patient 3)’, ‘A box of Treprostinil costs 10,000 RMB. I need to be rechecked frequently because the doctor needs to adjust the dose according to my condition. This means that in addition to expensive medicine, there are many additional costs, such as transportation (patient 9)’, ‘I stopped taking my medicine because I couldn't pay for it. Obviously, I felt more and more shortness of breath after stopping the drug (patient 19)’, ‘The operation was costly (patient 12)’, ‘I can't afford the operation anymore. I'm going to tell the doctor tomorrow that I refuse the operation (patient 7)’.*

### Theme 2: Fear and uncertainty about the disease

#### Psychological trauma following operations

Participants said frequent surgeries failed to cure the disease, leaving them feeling tired and losing faith in treatment Patients may feel fatigued from frequent operations and gradually lose confidence in treatment. *‘Internet information indicates that CTEPH is the most likely type of pulmonary hypertension to be cured with surgery. There was a time when I was full of expectations. However, I've had five surgeries in the past year, and each time I've always been one step away from success. The constant surgery makes me tired (patient 1)’, ‘When will it be completely cured? I don't think anyone can answer the question. After the surgery, the symptoms are obviously alleviated, but the next time I go to the hospital, the doctor may tell me that some indicators have changed and I must have the surgery again. I think maybe there is no complete cure, only constant "mending" can keep me alive (patient 15)’.*

#### Disease information is difficult to obtain

Some participants complained that the disease information reported on the Internet was numerous and inaccurate, and it was difficult to obtain effective information. Insufficient information regarding nursing care and symptom management can lead to anxiety and panic. Participants express their expectation of getting support from medical professionals.* ‘I am not sure whether the information on Baidu is correct, which makes me very upset (patient 3)’, ‘Every morning I take a deep breath to exercise lung function, but I feel more and more breathlessness. I would like to consult the doctor about whether this type of exercise is wrong and how I should exercise (patient 6)’.*

#### Feeling of fear and uncertain

Patients feel fear and uncertain about the disease which further affect the physical and mental health of patients. *‘When I was short of breath, I felt terrible and i am afraid that I will die (patient 18)’, ‘Every day effected by the disease, lying in bed with pain, living is so hard and painful, it is better to die, so as relief from this crime (patient 13)’, ‘I have a bad feeling that I don't know when my life will suddenly end (patient 4)’, ‘At night, my heart was beating very fast. I was flustered and scared. I do not dare to sleep. Even I am sleepy, I keep awake. I am very helpless (patient 4)’, ‘I was very sick, and I was so scared (patient 5)’.*

### Themes 3. Frustration in social and family role

#### Interference with work, school, and future plans

The disease was a disruption in the lives of patients with PH, especially in younger patients, which make them feel low self-esteem, unwillingness and uncertainty due to limited self-realization. ‘*Recently, I feel my health is deteriorating and I may not be able to go to clinical practice. I also want to pursue a master's degree, but my poor physical condition may not be able to support intensive studies (patient 4)’, ‘Due to lack of energy, I couldn't stick to my studies. After one hospitalization, I decided to stop going to school (patient 20)’, ‘At school, I seem to have a lot of privileges. Because I was so tired every day, I had to sleep in class, and the teacher never scolded me (patient 16)’.*

Some participants reported reduced productivity and efficiency at work due to lack of energy, and busy work also contributed to poor physical condition. ‘*I used to work as a casher in a supermarket, and I had to stand at a fixed station all day. I felt very tired when I came back home after work. One day I suddenly felt chest tightness, suffocated, and soon fainted. As a result, I realized that my physical condition was getting worse, and I quit my job and came home to rest (patient 3)’.*

#### The weakened function of family role

Disease depriving female patients of the right to breastfeed and also interferes with the patient's role as a mother in the child's growing path. The great conflict between the role of the patient and the role of the mother makes the patient feel guilty. *‘When my daughter was born, I was so sick time that I couldn't hold her. She was forced to wean early because I needed to take medication. After my daughter entered kindergarten, I was unable to participate in parent-child activities. My daughter is so poor. I feel guilty (patient 9)’.*

Participants often felt the emotional burden when they were unable to take on their household responsibilities of housekeeping and the care of children due to their physical conditions. ‘*Mopping the floor exhausts me. I drag a room and need to stop for a while. I get short of breath when I walk too long, and I can't carry heavy shopping bags, so my husband takes on the responsibility of shopping (patient 12)’, ‘I can't take care of my grandchild. Instead, my daughter has to take care of me. I put living burden on my daughter (patient 19)’.*

Younger Participants reported that they refuse to get married because they inability to take on family responsibilities. ‘*How can I take care of my family when I can't take care of myself? (patient 4)’, ‘I envy my friend for having a boyfriend, but I refuse to get married, and I know it sounds contradictory. I'm not allowed to have a baby because of my heart condition. You know, it's a sure recipe for family conflict (patient 20)’.*

Participants said they wanted to become mothers but feared the risks of pregnancy, and that not being able to conceive because of illness put pressure on their marriages. ‘*I got divorced because I am unable to get pregnant again due to disease (patient 10)’,* ‘*I wanted to be a mother, but the risks associated with pregnancy were terrifying. My husband suggested me to adopt a child (patient 3)’.*

### Theme 4: Lack of recognition of the condition

#### Loss of confidence in social activities

Patients passively stay away from social interaction due to their cardiopulmonary function, or to secure themselves in self-space. ‘*Every day, I stay at home alone. My relatives have their own lives, so they can't visit me every day. I am eager to talk to others. I yearn for company (patient 2)’, ‘When I am in a bad mood, I will lock myself in my room. I would rather listen to music alone than talk with others, including my parents. I have only two friends and I don't want to disturb their lives all the time. Before, I also tried to add a patient communication group on Wechat, but I did not know how to communicate with them (patient 20)’, ‘Apart from work, I just want to stay at home alone (patient 14)’.*

#### Restrictions on leisure activities

In order to avoid exertional dyspnea, patients usually minimize and limit leisure activities as much as possible. ‘*I haven't played with my friends since I got sick. I am eager to play basketball. Now my only exercise is to go for a walk with my dad, because the doctor told me to do proper activities, which can improve my heart and lung function (patient 16)’, ‘I like swimming very much, but now, the doctor advised me to stop it (patient 15)’, ‘I haven't traveled for a long time. I'm afraid I'll get worse if I'm too tired during the trip (patient 17)’.*

#### Lack of PH knowledge

It is difficult for patients to acquire effective disease management knowledge because of the single way of acquiring scientific disease knowledge and the mixed and inaccurate knowledge of disease popularization on the internet. The lack of disease knowledge can easily cause patients to be agitated, anxious and panic. ‘*I often go to Baidu on the internet to see what Kuaishou explains. I ask in the patient group, but there are so many different kinds of information. I don't know if it's true, but if I look at it for a long time, I'll get very upset (patient 3)’. ‘I read on the internet that my disease is hereditary. I don't know if it's true, but I'm a little scared (patient 7)’, ‘I take a deep breath every morning to exercise my lung function, but I feel that I'm getting more and more suffocated (patient 6)’.*

#### Impact of the COVID-19 epidemic

Participants reported that they experienced medication shortages and difficulty in seeing a doctor due to city blockade and traffic controls during the COVID-19 outbreak. Importantly, there is no effective solution and social support. ‘*I can't go to the doctor because of the city blockade. Even if I could get out, I was worried about contracting COVID-19 on the way to the hospital. What makes me anxious is that I was experiencing medication shortages, as pharmaceutical factories were unable to deliver medicines to hospitals because of the city lockdown and the temporary cessation of express delivery (patient 9)’.*

## Discussion

This is the first study which presents the patients subjective psychological experiences of living with PH in China. Despite treatment advances improving survival, patients with PH present with a range of debilitating symptoms, these experiences cause them to have some perceptions about their roles, physical status, and economic burden they faced which bring them with tremendous psychological challenges. Four main obtained themes of this study included “burden of PH treatment”, “fear and uncertainty about the disease”, “frustration in social and family role”, and “lack of recognition of the condition” reflect psychological experiences of patients living with PH.

A range of perplexed symptoms were experienced by participants including, dyspnea, chest tightness, chest pain, fatigue, edema and other symptoms, resulting in severe decline in activity tolerance, fear and uncertainty about the disease process and self-consciousness, consisted with other study [24, 25]. Due to the onset of symptoms and pain, our interviewees expressed ‘fear’ of engaging in activities which was closely interlinked with negative psychology. Despite the advent of PH therapies, the time from symptom onset to PH diagnosis remains at >2 years [26]. As a consequence of progressive pulmonary vasculopathy, most participates present with advanced disease. Thus, it is vital to decrease the time to diagnosis of PH which enable treatment at an earlier stage when therapies may be more effective [27]. Meanwhile, encourage patient to participate in support groups whereas individuals could seek validation and normalization of their symptoms from people with shared experience and also look to help others [28, 29]. In addition, sleep disorder is common in our interviewees with most of which caused by nocturnal dyspnea, cough, hemoptysis, palpitation or side effects of targeted drugs. Previous studies also reported higher sleep disorders among PH patients [30]. Since sleep disorders, in turn, can lead to dysfunction of the cardiovascular system [31], psychological distress, and lower health-related quality of life in PH [30](Matura et al., 2015), further exacerbating the severity of the disease [32]. We suggest that sleep disturbance should be taken as a sign of health problems, and screening patients' sleep problems is helpful to implement early intervention.

Patients with PH are more vulnerable than peers to loneliness and social isolation in our study, which may due to poor cardiopulmonary function, decreased activity endurance, reduced social capacity, lack of companionship and care of relatives and friends. Participants reported work, study and leisure difficulties largely due to fatigue and shortness of breath. Also, the COVID-19 pandemic has exacerbated this phenomenon [33]. Individuals in similar studies also reduced or ceased many activities [34]. Reflected in this, was the feeling of restriction, the nature of which had a considerable impact on patients’ profession, family and social roles [35]. Six interviewees in our study indicated that the disease had changed their life trajectory, and brought serious impacts on their studies, work, family, social life, marriage and child-bearing, ultimately leading to a state of social isolation. US and European surveys have also demonstrated the profound detrimental impact of PH on social life, physical and emotional well-being, sexual relationships and ability to work [24, 36]. The patients in our study experienced psychological states of anxiety, worthlessness, uncertainty about the future, and hopelessness. Moreover, the previous survey data by our team reported 70.09% of depressive symptoms among participates [21], all of these experiences may result in a poor quality of life among them. Patients in our study reported feelings of lost confidence and hope for the future which may impact their ability to adapt to the challenges of PH. From this view, it is clear that the impact of PH on patients’ psychology is far greater than its impact on physical condition. Although evidences pointed out the importance of psychiatric intervention to patients with PH [35]. But not only in our previous quantitative studies [21], but also in other studies, the number of patients receiving psychiatric treatment was very low [37, 38] . The reason may be varied [20], it further indicates that there may be a place in PH management by addressing physical, intellectual, emotional, social and spiritual needs. Therefore, the treatment of patients with PH should not only focus on the extension of survival, but also patients mental health and the quality of life. The psychological support should not only rely on psychological regulation and drug treatment, but also mobilize social support to meet the needs of patients’ self-realization.

PH treatment is challenging for patients, especially when it comes to involving surgery. Pulmonary endarterectomy is the only curative therapy for CTEPH, however, approximately 40% of patients are not eligible for this intervention [39], in which balloon pulmonary angioplasty can be performed instead to provide adequate treatment for affected pulmonary arteries. Balloon pulmonary angioplasty vary in frequency from 3 to 10 operations depending on the condition of the vessel being occluded [8]. Patients were fatigued due to frequent operations, gradually lost confidence in treatment in our study. Study shows that for every additional balloon angioplasty, patients experience an annual increase in hospitalization costs of $10,000 and drug costs of $20,000 [40]. Moreover, patients need long-term postoperative anticoagulation drugs, together with targeted drug. All which place a heavy financial burden on patients. In addition, studies have found that hemodynamics improvement from the third time balloon angioplasty will be attenuated, leading to an illusion of surgical futility [41]. Our interviewees indicated that economic pressure, repeated illness, multiple hospitalizations, high cost of targeted drugs and surgery cause unaffordable treatment burdens, especially for those with low-income, leading them to stop the medicine or refuse the surgery. Non-adherence to PH-specific medications has been reported in around 25–40% of patients across different studies [42]. We did not deeper the real reasons for non-adherence in PH which could be complex. However, the consequences for failing to take medication can be life-threatening [43]. This also indicates that the patients’ financial capabilities and the availability of medical resources have an impact on how the patients cope with the disease. Given the potentially consequences, early, comprehensive, and ongoing education is critical to convey the seriousness of the disease and the importance of adhering to recommended treatment [44].

In addition, the results of the interviews showed that the patients had insufficient objective information support, less access to disease information and lack of disease knowledge. Due to increased number of PH cases and resource constraints in health care systems, health policymakers and other community members must provide a comprehensive support for patients with PH, such as up-to-date information about disease and care, encourages them to continue their treatment. Health care staffs need to be clear about what they can do to help patients and also work to strengthen the sense of self-efficacy and empowerment. Due to limited physical contact, a telemedicine support service should be developed for patients’ education and consultation. Using telephone-based follow-up and web-based technology with features such as educational posters and videos and online chat sessions may be effective strategies to facilitate efficient and effective cares.

There are some limitations in this study. The subjects of this study are from one hospital, and the representativeness of the study results is affected to some extent. In addition, our study subjects were mainly hospitalized patients, physical and psychological symptoms may be worse.

## Conclusions

In summary, this interview shows that PH brings great burden to patients regarding their physical, psychological, and social aspects, leading widespread impact on many aspects of patients’ lives. In the face of such a rare disease, patients are facing with challenges in terms of an understanding of the disease, and treatment adherence. Patients and healthcare professionals must work in partnership to identify and implement individualized and holistic management approaches for optimal outcomes that are meaningful for patients.

## Relevance for clinical practice

This study provides healthcare professionals with information on the life burden and mental health problem of patients with PH. The findings of this study support the further development of clinical practices to improve care and support of people with PH. We recommend that healthcare professionals develop assistance services, including disease management programs, psychological support services, mobilisation of social support, and telemedicine services.

## Data Availability

The datasets used and/or analysed during the current study are in Chinese and are available from the corresponding author on reasonable request but will require translation to English.
